# Contrast Agents for Photoacoustic and Thermoacoustic Imaging: A Review

**DOI:** 10.3390/ijms151223616

**Published:** 2014-12-18

**Authors:** Dan Wu, Lin Huang, Max S. Jiang, Huabei Jiang

**Affiliations:** 1 School of Physical Electronics, University of Electronic Science and Technology of China, Chengdu 610054, China; E-Mails: uestcabil@163.com (D.W.); ilyzlhl@163.com (L.H.); 2 College of Medicine, University of Central Florida, Orlando, FL 32827, USA; E-Mail: themaxj123@gmail.com; 3 Department of Biomedical Engineering, University of Florida, Gainesville, FL 32611, USA

**Keywords:** photoacoustic imaging, thermoacoustic imaging, molecular imaging, contrast agents

## Abstract

Photoacoustic imaging (PAI) and thermoacoustic imaging (TAI) are two emerging biomedical imaging techniques that both utilize ultrasonic signals as an information carrier. Unique advantages of PAI and TAI are their abilities to provide high resolution functional information such as hemoglobin and blood oxygenation and tissue dielectric properties relevant to physiology and pathology. These two methods, however, may have a limited detection depth and lack of endogenous contrast. An exogenous contrast agent is often needed to effectively resolve these problems. Such agents are able to greatly enhance the imaging contrast and potentially break through the imaging depth limit. Furthermore, a receptor-targeted contrast agent could trace the molecular and cellular biological processes in tissues. Thus, photoacoustic and thermoacoustic molecular imaging can be outstanding tools for early diagnosis, precise lesion localization, and molecular typing of various diseases. The agents also could be used for therapy in conjugation with drugs or in photothermal therapy, where it functions as an enhancer for the integration of diagnosis and therapy. In this article, we present a detailed review about various exogenous contrast agents for photoacoustic and thermoacoustic molecular imaging. In addition, challenges and future directions of photoacoustic and thermoacoustic molecular imaging in the field of translational medicine are also discussed.

## 1. Introduction

Photoacoustic imaging (PAI) is a burgeoning biomedical structural and functional imaging technique which utilizes ultrasonic signals as an information carrier that captures information about optical absorption property relevant physiology and pathology inside tissues. The basic process of the PAI excitation source is summarized below: (1) the tissue absorbs light energy after being irradiated by visible light or near-infrared light; (2) the absorber produces adiabatic expansion that induces photoacoustic signals [1]; (3) the transducers detect the signals; (4) a data acquisition card stores up information for image reconstruction analysis. PAI has a high contrast and resolution for the combination of excellent selectivity of optical imaging and high penetration of ultrasonic imaging. The image resolution and maximum imaging depth can be adjusted with the ultrasonic frequency and the penetration of diffuse photons. PAI has a close relationship with optical properties, thermal properties and acoustic properties of biological tissue. PAI incorporates biochemical information, physical characteristics, metabolic status, pathological changes and even neural activity because the spectrum of tissue is related to the molecular structure. For instance, PAI is suitable for applications in vascular structural and functional imaging or tumor imaging due to the ability to acquire hemoglobin information. So far, several variations of photoacoustic imaging have been developed, including Photoacoustic Spectroscopy (PAS), Photoacoustic Tomography (PAT), Photoacoustic Microscopy (PAM), and Photoacoustic Endoscopic imaging (PAE) [2,3].

Thermoacoustic Imaging (TAI) is another emerging biomedical imaging technique. Broadly defined, TAI is an application of the photoacoustic effect. Unlike PAI, a TAI excitation source involves far-infrared light or microwaves. It also provides longer imaging depth due to a different electromagnetic radiation. TAI offers higher spatial resolution than microwave imaging and receives much deeper imaging than most optical imaging techniques [4]. Since it is based on a different absorption mechanism, TAI can capture information about dielectric properties (such as the distributional difference of some polar molecules and ions) of the relevant physiology and pathology inside of tissues. TAI could potentially be used for early cancer detection and foreign bodies’ detection [5]. Therefore, the advantages of PAI and TAI over other imaging modalities will be likely to make the application of these two techniques in clinical medicine more feasible and practical.

There are several major endogenous contrast agents used in PAI, including hemoglobin, melanin, lipid and water in tissues. TAI is usually performed by using the distributional difference of water content and ions’ concentration. In the situation where a limited detection depth is certain or an endogenous contrast is not available, an exogenous contrast agent can be utilized to effectively resolve these problems. Oral or injectable chemicals, known as exogenous contrast agents, can be used to enhance images in medical scans of the tissues or organs. Besides improve resolutions, they can also potentially break through the imaging depth limit. Furthermore, a receptor-targeted contrast agent can trace the molecular and cellular biological processes in tissues. Thus, photoacoustic and thermoacoustic imaging can be outstanding tools for early diagnosis, precise lesion localization and molecular typing of various diseases. A targeted contrast agent can also be used for therapy in conjugation with drugs or photothermal therapy, and the double virtues of imaging and targeted therapy will then be possessed.

## 2. Exogenous Contrast Agents

With the development of novel imaging technologies, contrast agents have played an important role in molecular imaging. There are various principles, materials, shapes, and sizes of contrast agents suitable for different imaging modalities. Ideal contrast agents could significantly increase contrasts, effectively improve imaging depth or accuracy, and provide molecular specific information. For instance, systemic delivery of the nanoprobe into the mice bearing mammary tumors led to a significant improvement in the photoacoustic signals and enabled PAI of tumors located as deep as 31 mm beneath the tissue surface [6]. The recent surge of interest has expanded the depth of TAI techniques with exogenous contrast agents to biomedical imaging, such as breast cancer imaging, foreign body detection, and targeted tumor detection [4]. Some contrast agents have a passive-targeting ability, that is, they can extravasate into tumor tissues because of impaired vasculature and the enhanced permeability and retention (EPR) effect in solid tumors. Other contrast agents conjugated with a molecular probe (such as antibodies, proteins, nucleic acids and peptides) possess an active-targeting ability. As ligands, they bind to a specific subset of receptors in target cells or tissues. They may also act as drug carriers by significantly enhancing the drug delivery to lesions. In such a role, the efficacy of the drug is improved while the side effects are reduced due to substantial alterations in the drug’s distribution and metabolism. From a translational standpoint, further research on exogenous contrast agents is of great importance in order to transform photoacoustic and thermoacoustic molecular imaging research into clinical applications.

### 2.1. Contrast Agents for PAI

In this section, a detailed review about various exogenous contrast agents for photoacoustic molecular imaging is presented. In Table 1, they are divided into five types, which are classified by principle and function, including dyes, plasmonic nanoparticles, various new class nanoparticles, multimodality contrast agents, and theranostic contrast agents.

#### 2.1.1. Dyes

Dyes are the first class of photoacoustic contrast. Most of them are nanometer scale fluorescent molecules which can be readily removed by the urinary system. Indocyanine-green (ICG), for example, is an FDA approved fluorescent contrast agent that can be used for both fluorescence and photoacoustic imaging. It absorbs light primarily in the range of 600–900 nm and emits fluorescence light from 750–950 nm. Wang, *et al.*, utilized ICG of various concentrations, sizes, and depth locations, along with PAT and Fluorescence Molecular Tomography (FMT) in an experiment involving a target embedded in a background phantom. Their results found that when ICG is administered in combination with high resolution PAT and high sensitivity FMT, a better diagnostic tool is created. The efficiency of a contrast agent coupled with these tools therefore demonstrates a potential for clinical applications in the future [7]. A second dye that functions as an effective contrast agent is Alexa Fluor 750, a common near-IR fluoescent dye. Razansky, *et al.*, showed that the depth-resolved distribution of flurochromes, Alexa Fluor 750, in small animals can be imaged with 25 fmol sensitivity and 150 μm spatial resolution by multispectral photoacoustic imaging [8,9]. Another effective agent, Evans blue contrast dye, was imaged by Li, *et al.*, in order to see its wash-in process in cortex vasculature. This dye can tightly bind to serum albumin in the vasculature and has been used to determine blood volume in a study of the blood-brain barrier [10]. Finally, Yang, *et al.*, developed a novel near-infrared dye-based imaging caspase-9 probe that directly detects apoptosis with a high specificity in cancer cells by PAI [11]. It should be noted that there are many other dyes used in PAI, including BHQ3, QXL680 [12], IRDye800CW [13], MMPSense™ 680 [14] and Methylene blue [15].

**Table 1:** Contrast agents for photoacoustic imaging.

Photoacoustic Contrast Agent	Type	Absorption Peak (nm)	Size (nm)	Modification Application	Application	Ref.
Indocyanine-green	NIR Fluorescent Dye	810	<2	CarbonNanotube, PEG, PEBBLEs	PAT, in tissue phantoms and *in vivo*	[7,16,17,18,19]
Methylene blue	NIR Fluorescent Dye	650–700	<2		PAT, in tissue phantoms	[15]
Alexa Fluor 750	NIR Fluorescent Dye	750	<2		Multispectral PAI, *in vivo*	[8,9]
IRDye800CW	NIR Fluorescent Dye	750–800	<2	NPR-1	PAS, *in vivo*	[13]
IRDye800-c(KRGDf)	NIR Fluorescent Dye	750–790	<2	Integral proteinαvβ3	PAS, *in vivo*	[20]
Evans Blue	NIR Fluorescent Dye	550	<2		PAT, *in vivo*	[10]
PPCy-C8	NIR Fluorescent Dye	754–789	<2	Perfluorocarbon	*In vivo*, dual-modality PAI-FI	[21]
Cypate-C18	NIR Fluorescent Dye	754–790	<2	Perfluorocarbon	*In vivo*, dual-modality PAI-FI	[21]
Caspase-9 Probe	NIR Fluorescent Dye	640	<2		PAI, *in vivo*	[11]
MMPSence™ 680	NIR Fluorescent Dye	620, 680	<2		PAI, in tissue phantoms	[14]
BHQ3	Quencher	672	<2		PAI, *in vitro*	[12]
QXL680	Quencher	680	<2		PAI, *in vitro*	[12]
Au Nanospheres	Plasmonic Noble Metal Nanoparticle	520–550	20–80	PEG	PAT, *in vivo*	[22,23]
Au Nanoshells	Plasmonic Noble Metal Nanoparticle	700–1100	50–500	PEG	PAT, *in vivo*	[24,25]
Au Nanorods	Plasmonic Noble Metal Nanoparticle	550–1550	a few to hundreds of	HER2, EGFR	PAI, *in vitro*	[26,27,28]
Au Nanocages	Plasmonic Noble Metal Nanoparticle/Theranostic Contrast Agent	820	25		PAT, *in vivo*, photothermal therapy	[29,30,31]
Au Nanoclusters	Plasmonic Noble Metal Nanoparticle	500–550	100		PAI, *in vitro*	[32,33]
Au Nanostars	Plasmonic Noble Metal Nanoparticle	767	120		PAT, *in vivo*	[34,35]
Au Nanobeacons	Plasmonic Noble Metal Nanoparticle	520	150	α_v_β_3_	PAT, *in vivo*	[36,37]
Ag Nanoplates	Plasmonic Noble Metal Nanoparticle	550–1080	25–218	a-EGFR, PEG	PAI, *in vivo*	[38]
Ag Nanosystems	Plasmonic Noble Metal Nanoparticle/Theranostic Contrast Agent	400–500	180–520		PAI, *ex vivo*; image-guided therapy	[39]
Quantum dots	Nanoparticles Based On Other Principles	400–750	<10		PAT, *in vivo*: Triple-modality PA-PT-Fluorescent	[40]
Nanodiamond	Nanoparticles Based On Other Principles	820	68.7		PAI, *in vivo*	[41]
Polypyrrole Nanoparticles	Nanoparticles Based On Other Principles	700–900	46		PAI, *in vivo*	[42]
Copper Sulfide	Nanoparticles Based On Other Principles	900	11 ± 3		PAI, *in vivo*	[43]
Graphene Nanosheets	Nanoparticles Based On Other Principles	200–900	10		PAI, *in vitro*	[44]
Iron Oxide-gold Core-shell	Multimodality Contrast Agent	660–900	1–5		Triple-modality MRI-PAI-mmPA	[45]
Gd_2_O_3_	Multimodality Contrast Agent		100	DEG, gelatin	*In vivo*, dual-modality PAT-MRI	[46]
Single-walled Carbon Nanotubes (SWNT)	Multimodality Contrast Agent	785	5–8	Protamine, PEG	*In vivo*, Triple-modality Raman- MRI-PAI	[47]
Dye-loaded Perfluorocarbon-based Nanoparticles	Multimodality Contrast Agent	750–800	220 ± 11	cypate-C18, PPCy-C8,PEG2000, phosphatidylethanolamine	*In vivo*, dual-modality PAI-FI	[21]
AuMBs	Multimodality Contrast Agent	760	100–1000	HAS	Dual-modality PAI-UI	[48]
Triggered Nanodroplets	Multimodality Contrast Agent	750–800	300	Perfluorocarbon	In tissue phantoms and *in vivo*, dual-modality PAT-UI	[49]
Cobalt Nanowontons	Multimodality Contrast Agent	700	30–90		Dual-modality MRI-PAT	[50]
Nanoroses	Multimodality Contrast Agent	700–850	30		PAI, *in vitro*	[51]
MPRs	Theranostic/Multimodality Contrast Agent	532	120	maleimide-DOTA-Gd	*In vivo*, triple-modality MRI-API-Raman; image-guided surgery	[52]
Goldsilica Core shell Nanorods	Theranostic Contrast Agent	780	10.3 ± 1.1	PEG	PAI, *in vitro*	[53,54]
Superparamagnetic Iron Oxide (SPIO)	Theranostic Contrast Agent	500–780	80–150		PAI, *ex vivo*	[55]

Dyes can also be surface-modified or conjugated with other contrast agents. Li, *et al.*, used PAS to obtain high resolution images of the distribution of IRDye800-c(KRGDf) targeting integrin α_v_β_3_ overexpressed in human U87 glioblastomas in nude mouse brains. Simultaneously, the hemoglobin oxygen saturation and the total hemoglobin concentration of the vasculature which revealed hypoxia in tumor neovasculature were obtained [20]. Akers, *et al.*, explored perfluorocarbon-based nanoparticles by incorporating two different NIR fluorescent dyes, PPCy-C8 and cypate-C18, as PA and fluorescence imaging agents. They demonstrated the contrast agent’s ability of lymph node mapping with both modalities [21]. Besides these surface-modified dyes, there are many other dyes developed for PAI, such as Indocyanine-green-embedded PEBBLEs [16], ICG-PEG [17], ICG-enhanced carbon nanotubes [18], and ICG encapsulated in virus-mimicking nanoconstructs [19].

#### 2.1.2. Plasmonic Noble Metal Nanoparticles

Noble metal nanoparticles have been widely used as photoacoustic contrast agents due to their intrinsic optical absorption, surface plasmon resonance (SPR), effective surface modification, and high technical maturity of preparation. It is well-known that the plasmon resonance of metal nanoparticles is highly sensitive to the nanoparticle size, shape, and dielectric properties of the surrounding medium [56,57]. Usually, the optical absorption of noble metal nanoparticles is a few orders of magnitude larger than that of traditional dyes. There are essentially two types of noble metal nanoparticles used in PAI: gold nanoparticles and silver nanoparticles.

**Figure 1:**
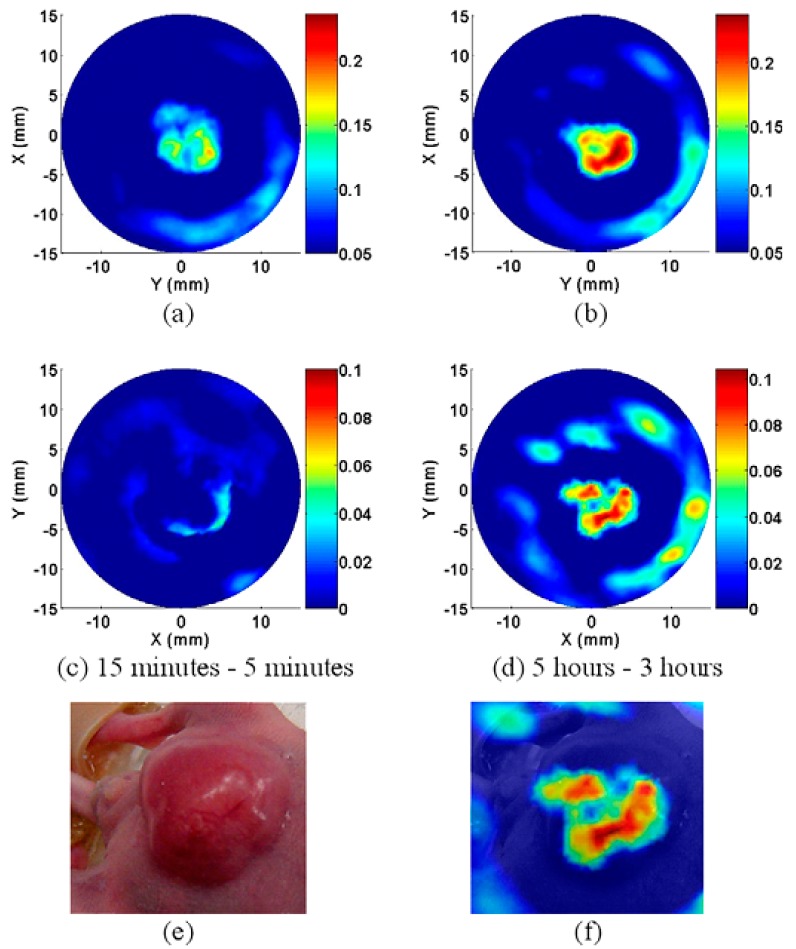
PAT images of tumor at 5 min (**a**) and 5 h (**b**) following tail vein injection of gold nanoparticles; (**c**) and (**d**) are the subtraction PAT images of tumor following tail vein injection of gold nanoparticles demonstrating increased accumulation of nanoparticles in tumor at 5 h. The color scale (right) represents optical absorption of tissue (arbitrary units); (**e**) is gross picture of tumor in mouse and (**f**) is the fusion image of gross photo and subtraction PAT image, 5 h following tail vein injection. (Reprinted from reference [22]. Copyright with permissionfrom © 2009 IOP Publishing Ltd.).

**Figure 2:**
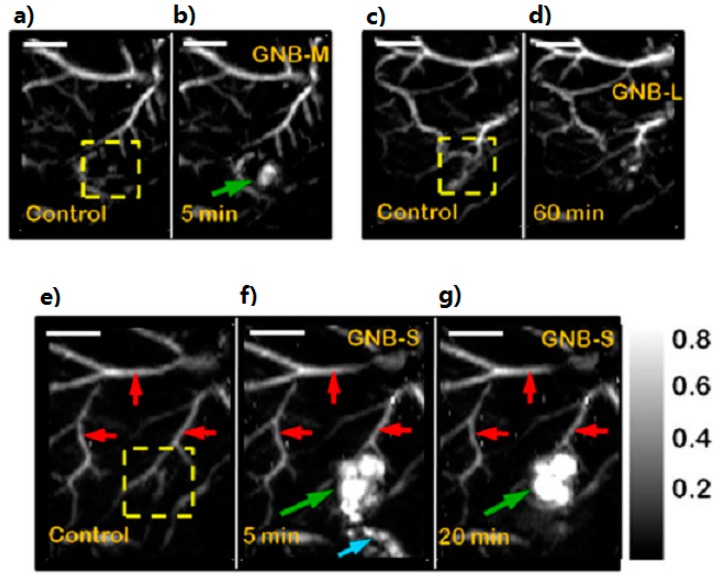
*In vivo* noninvasive photoacoustic imaging of sentinel lymph nodes in rat (λ = 767 nm). (**a**–**g**) Scale bar is 5 mm. Aliquots of 150 mL of nanobeacons were injected intradermally in all cases. GNB-M: (**a**) control PA image; (**b**) 5 min post-injection image of GNB-M (5 mM); GNB-L: (**c**) control PA image; (**d**) lymph node is not visible in a 60 min post-injection image of GNB-L (680 nM); GNB-S: (**e**) sagittal maximum amplitude projection (MAP) pre-injection control image; bright parts represent optical absorption from blood vessels, marked with red arrows; (**f**) PA image (MAP) acquired 5 min after GNB-S injection (10 nM); SLNs are clearly visible, marked with green arrows; lymphatic vessel is also visible, marked with blue arrows; (**g**) 20 min post-injection PA image. (Reprinted from reference [37]. Copyright with permission from © 2011 John Wiley & Sons, Ltd.).

The optical properties of gold nanoparticles can be readily tuned by varying their size and shape. Their relative scattering to absorption contribution could be easily tuned by a change in their dimensions. There are a trend that larger nanoparticles would be more suitable for biological cell imaging applications based on light scattering, while those in the intermediate size range would generate more optical-to-acoustic conversion and then serve as excellent photoabsorbers for laser photothermal therapy and applications based on absorption contrast [57]. The optical-to-acoustic conversion efficiency represents how many incident photons will be absorbed and converted to heat and how fast this heat can diffuse from the target during thermoelastic expansion and wave generation. As such, this conversion efficiency will determine the contrast intensity of photoacoustic imaging [6]. These nanoparticles thus exist in a variety of shapes and sizes: Au nanosphere, Au nanoshell, Au nanorod, Au nanocage, Au nanocluster, Au nanostar, Au nanobeacon, andso on. To begin with, Au nanospheres consist of one important categorization of nanoparticles. The common size range employed (approximately 40 nm) is able to display an absorption cross-section five orders higher than any conventional absorbing dyes. In contrast, the magnitude of light scattering by 80-nm gold nanospheres is 5 orders higher than light emissions from strongly fluorescing dyes. It can be seen then that absorption wavelength will increase with size. Unfortunately, the plasma resonance absorption peak of nanospheres (520–550 nm) is too limited to be of any use for *in vivo* applications [57,58,59]. However, Zhang, *et al.*, have evaluated the ability of systemically administered poly(ethylene glycol)-coated (PEGylated) gold nanoparticles as a contrast agent for *in vivo* tumor imaging with PAT. They have properly demonstrated that the accumulation of gold nanoparticles in tumors, due to EPR effects, can be efficiently imaged with PAT. These experiments were conducted via IV administration of these nanoparticles into tumor-bearing mice (as shown in Figure 1) [22,23]. The next classification involves the Au nanoshell, which is an optically tunable nanoparticle consisting of a dielectric core (silica) surrounded by a thin metallic layer (gold). Au nanoshells have optical cross-sections comparable to and even higher than Au nanospheres. By increasing the total nanoshell size or the ratio of the core-to-shell radius, the resonance wavelength of nanoshells can be precisely and systemically controlled over a broad spectrum, including the near-infrared region where optical transmission occurs through biological tissues.Wang, *et al.*, has used PAT to image *in vivo* distributions of poly(ethylene glycol)-coated nanoshells circulating in the vasculature of a rat brain. Their results show that optical absorption in the brain vessels was enhanced by up to 63% after three sequential administrations of such nanoshells [24,25]. Third, Au nanorods are yet example of an excellent photoacoustic contrast agent. They are able to display optical cross-sections comparable to nanospheres and nanoshells, however, at a much smaller effective size. Their optical resonance can be linearly tuned across the near-infrared region by changing either the effective size or aspect ratio of the nanorods. Gold nanorods show per micron absorption and scattering coefficients that are an order of magnitude higher than those of nanoshells and nanospheres [26,27]. Li, *et al.*, conducted studies involving HER2, EGFR, and CXCR4 as the primary target molecules binding to Au nanorods in order to examine two types of cancer cells, OECM1 and Cal27. OECM1 cells overexpressed HER2, but exhibited a low expression of EGFR, whereas Cal27 cells showed the opposite expression profile. Single and double targeting resulted in signal enhancements of up to 3 dB and up to 5 dB, respectively, and therefore a potential in improving cancer diagnoses has been portrayed [28]. Next, an Au nanocage is a type of cage-like multihollow optically tunable nanoparticles. Yang, *et al.*, were able to sequentially inject poly(ethylene glycol)-coated Au nanocages into the circulatory system of a rat in three administrations. *In vivo* PAT was conducted immediately prior to the first injection and performed continuously until 5 h after the final injection. Results show that a gradual enhancement of the optical absorption in the cerebral cortex, by up to 81%, was observed over the course of the experiment [29,30,31]. Shortly after the success of Yang, *et al.*, Yoon, *et al.*, developed a kind of photoacoustic contrast agents named “biodegradable nanoclusters”, which consist of sub-5 nm primary gold nanoparticles stabilized by small amountsof biodegradable polymer. The nanocluster assembly is controlled by a weakly adsorbing biodegradable polymer through a combination of electrostatic, and depletion forces [32,33]. The sixth category of noble metal nanoparticles involves star-shaped gold nanoparticles (“nanostars”). These stars have plasmon bands that are tunable into the NIR region. The structure contains multiple sharp branches that act as “lightning rods” that greatly enhance the local EM-field. The plasmon resonant wavelength correlates with the branching [34]. Kim, *et al.*, demonstrated that the high photoacoustic sensitivity of nanostars enable their *in vivo* detection in rat sentinel lymph nodes and vessels, suggesting a direct application toward lymph angiography [35]. Besides nanostars, gold nanobeacons (GNB) represent another effective contrast agent. Such beacons have a robust nanoparticle platform that entraps multiple copies of tiny gold nanoparticles (2–4 nm) within a larger colloidal particle encapsulated by biocompatible synthetic or natural amphilines. The utilization of numerous small gold particles significantly amplify the signal without exceeding the renal elimination threshold size (as shown in Figure 2) [36,37]. For the purposes of angiogenesis, an essential microanatomical biomarker of tumor and cardiovascular disease progression, integrin-targeted GNBs allows for visualization of numerous angiogenic sprouts and bridges.

**Figure 3:**
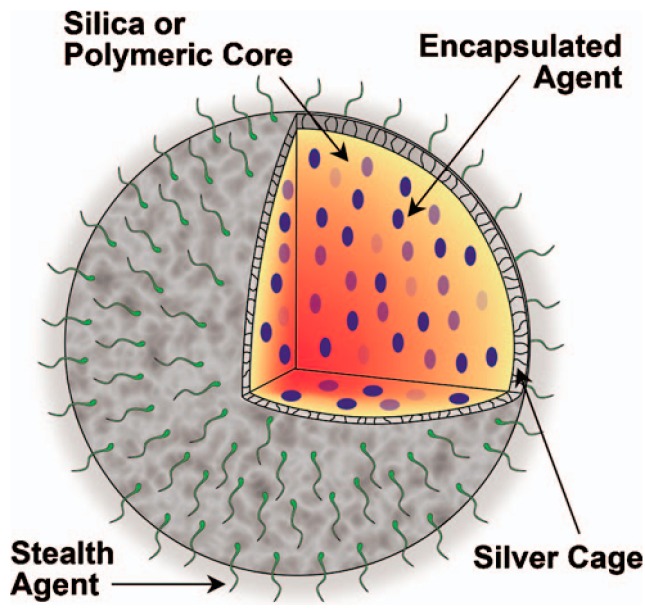
Multifunctional nanosystem platform capable of providing imaging contrast, drug delivery, and image-guided therapy. (Reprinted from reference [39]. Copyright with permission from © 2010 Society of Photo-Optical Instrumentation Engineers).

Finally, the last class of noble metal nanoparticles consists of silver nanoplates, which possess good stability, biocompatibility, and a low toxicity. Their use as a photoacoustic contrast agent can also be easily functionalized for molecular photoacoustic imaging *in vivo*. Once conjugated to a-EGFR, functionalized nanoplates underwent receptor-mediated endocytosis in pancreatic cancer cells that overexpress EGFR, demonstrating their potential for molecular specificity *in vitro* [38]. Similarly, a nanosystem consists of a porous silver layer deposited on the surface of spherical silica cores ranging in diameter from 180–520 nm (see in Figure 3). The porous nature of the silver layer allows for the release of drugs or other therapeutic agents encapsulated within the core for future applications. In their current PEGylated form, the silver nanosystem is shown to be nontoxic *in vitro* at concentrations of silver up to 2 mg/mL [39].

#### 2.1.3. Nanoparticles Based on Other Principles

Besides noble metal nanoparticles, there are others based on different principles. Quantum dot (QD) is an example of an exciting new class of nanoparticles composed of II-VI or III-V group elements. It has unique optical properties, such as relatively high quantum yields, broad excitation spectra ranging from ultraviolet to near-infrared, and relatively narrow emission spectra. It has been demonstrated that the application of QDs provide an opportunity for multimodal high resolution (300 nm) PA-PT-fluorescent imaging to flourish. Hybrid multilayer QDs, which have optimized absorption, thermal, and acoustic properties, may also be utilized in PT therapy. In this scenario, they function as enhancers of the conversion of laser energy in PT, PA, and bubble phenomena [40].

**Figure 4:**
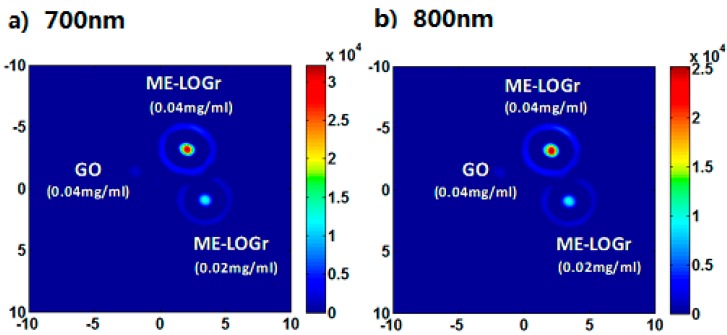
Photoacoustic (PA) signal of GO and ME-LOGr nanosheets of different concentrations, illuminated with 700 (**a**) and 800 (**b**) nm laser. The color coded vertical bar represents the strength of the photoacoustic signal generated. GO nanosheets were obtained via control-A experiment where nitronium ions and KMnO_4_ both act as an oxidant. (Reprinted from reference [44]. Copyright with permission from © 2013 American Chemical Society).

Radiation-damaged nanodiamonds (DNDs) are another set of potentially ideal optical contrast agents for PAI in biological tissues due to their low toxicity and high optical absorbance. In a set of studies, Zhang, *et al.*, were able to create new DNDs which produced a 71-fold higher PA signal on a molar basis in comparison to similarly dimensioned gold nanorods. 7.1 fmol of DNDs injected into rodents could be clearly imaged 3 mm below the skin surface with a PA signal enhancement of 567% [41]. A third type of nanoparticles involves polypyrrole nanoparticles (PPy NPs), novel organic PAT contrast agents. Monodisperse PPy NPs are about 46 nm in diameter with strong absorption in the near-infrared (NIR) range, which allows for visualization of PPy NP-containing agar gel embedded in a chicken breast muscle at a depth of about 4.3 cm. In comparison with PAT images based on the intrinsic optical contrast in mice, PAT images (within 1 h) following intravenous administration of PPy NPs showed the brain vasculature with greater clarity than hemoglobin in blood [42]. Next, semiconductor copper sulfide nanoparticles (CuS NPs) are also used effectively in PAI. The average diameter of a CuS NP was 11 ± 3 nm. The absorption peaked at around 990 nm. CuS NPs allowed for visualization of a mouse brain after intracranial injection, rat lymph nodes 12 mm below the skin after interstitial injection, and CuS NP-containing agarose gel embedded in chicken breast muscle at a depth of about 5 cm [43]. By intentionally excluding KMnO_4_ and exploiting pure nitronium ion oxidation, aided by the unique thermal and kinetic effects induced by microwave heating, graphite particles can be converted into microwave-enabled low oxygen grapheme (ME-LOGr) nanosheets with their π-conjugated aromatic structures and properties largely retained. Without the need of any postreduction processes to remove the high concentration of oxygenated groups that results from Hummers graphene oxide (GO) formation, the graphene nanosheets as-fabricated exhibited strong absorption and high photothermal. Patel, *et al.*, demonstrated that strong photoacoustic signals can be generated from these graphene nanosheets with NIR excitation (see in Figure 4). The photo-to-acoustic conversion is weakly dependent on the wavelength of the NIR excitation, which is different from all other photoacoustic contrast agents previously reported. From these results, it can be seen that these NPs possess great potential as nanocarriers to develop multifunctional drug delivery systems with on an “on demand” release. They may also be used for *in vivo* photoacoustic imaging capabilities for *in situ* evaluation of therapeutic effects and tracking their long term fate [44].

#### 2.1.4. Multimodality Contrast Agents

No mono-modality imaging can obtain all the information needed in biomedical imaging technologies. The preponderance complementarity of various imaging modes, multimodal imaging, will greatly improve medical high-tech. So far, there have been many imaging technologies used in combination with PAI. To begin with, ultrasound imaging (UI) has low test fees, is easy to operate, and can track in real-time. However, it has a low sensitivity and cannot detect lesions early due to its inability to distinguish adsorbers which have the same acoustic impedance but different dielectric properties. Next, magnetic resonance imaging (MRI) has a high resolution of soft tissues and maps macrostructure well, but is unable to differentiate benign from malignant tumours or track in real-time due to its long scan time. On the other hand, fluorescence imaging (FI) has a high sensitivity, can track in real-time and monitor dynamically, but cannot capture information in deep tissue because of a limited penetration; Raman imaging allows for highly specific and sensitive detection of surface-enhanced Raman scattering contrast agents, as well as the multiplexing of multiple agents in living subjects. Thus, the combination of PAI and examples of the above technology will prove to be a comprehensive source of information for structural and functional physiology and pathology.

**Figure 5:**
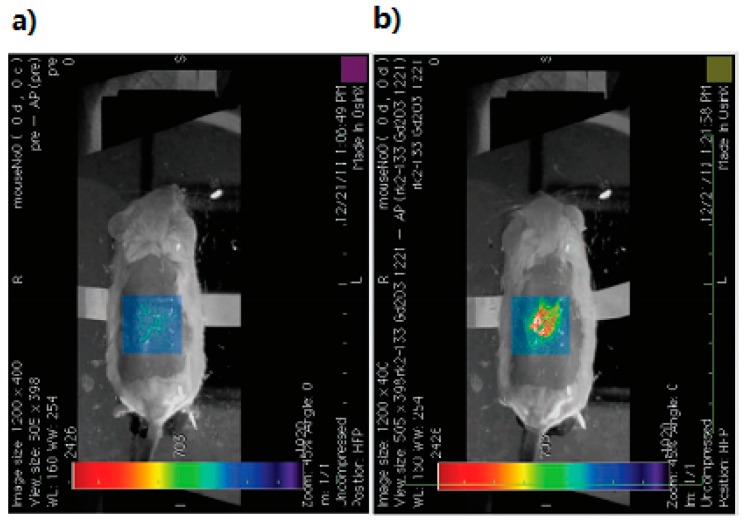
PAT-MRI images of tumor (**a**) before and (**b**) after injection of Gd_2_O_3_-DEG-gelatin. (Reprinted from reference [46]. Copyright with permission from © 2012 WILEY-VCH Verlag GmbH & Co. KGaA, Weinheim).

Although every individual imaging modality has its own contrast agents, multimodality imaging needs multimodality contrast agents. Magnetic nanoparticles are a category of such agents that have been widely applied in biomedicine. Gold-iron oxide composite nanoparticles, for example, open up vast possibilities to MRI-PAI dual-modal technology [6,60,61]. Jin *et al*., demonstrated that multifunctional iron oxide and gold-coupled core-shell nanoparticles (NPs), with well-defined structural characteristics and physical properties not only offer contrast for electron microscopy, MRI, and scattering-based imaging but, more importantly, enable a new imaging mode, magnetomotive photoacoustic imaging. This type of imaging displays a remarkable contrast enhancement compared with photoacoustic images using only conventional nanoparticles contrast agents [45]. Gold-speckled Gd_2_O_3_ nanoparticles and rare earth-doped Gd_2_O_3_ nanorods have also been used for optical and MR imaging. Kimura, *et al*., synthesized and isolated new size-controlled and biocompatible Gd_2_O_3_-DEG-gelatin nanoparticles as a bimodal contrast agent for use in PAI and MRI (see in Figure 5) [46].

**Figure 6:**
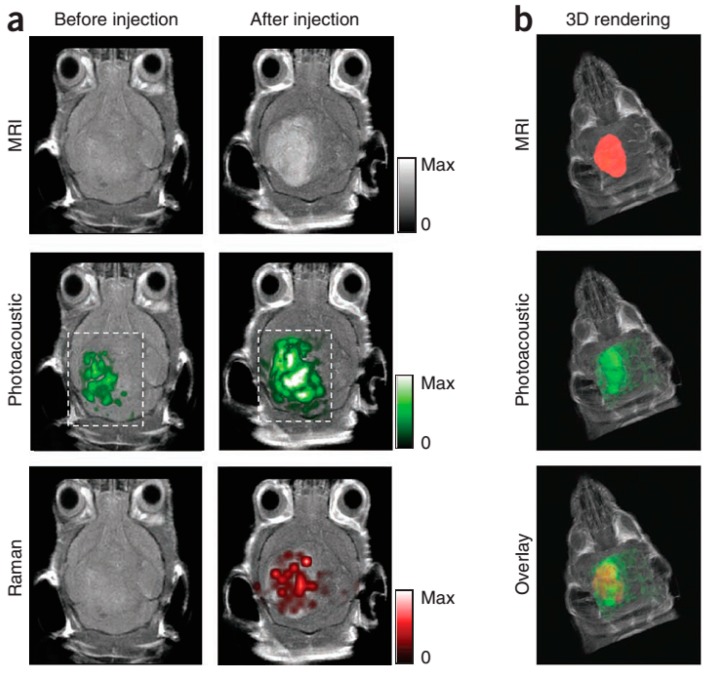
Triple-modality detection of brain tumors in living mice with MPRs. (**a**) Two-dimensional axial MRI, photoacoustic and Raman images. The post-injection images of all three modalities showed clear tumor visualization (dashed boxes outline the imaged area); (**b**) A three dimensional (3D) rendering of magnetic resonance images with the tumor segmented (red; **top**), an overlay of the three-dimensional photoacoustic images (green) over the MRI (**middle**) and an overlay of MRI, the segmented tumor and the photoacoustic images (**bottom**) showing good colocalization of the photoacoustic signal with the tumor. (Reprinted from reference [52]. Copyright with permission from © 2012, Rights Managed by Nature Publishing Group).

Another type of multimodality contrast agents are single-walled carbon nanotubes (SWNTs) which have broad absorption that can produce strong photoacoustic signals. They possess surface modification and biocompatibility similar to plasmonic nanoparticles. Wang, *et al.*, have used such SWNTs to develop a novel approach in labeling human mesenchymal stem cells (hMSCs) with polyethylene glycol (PEG) functionalized SWNTs conjugated with protamine (SWNT-PEG-PRO) for *in vivo* tracking by Raman-MRI-PAI triple-modal imaging [47]. Microbubbles (MBs), which can be composed of phospholipids, albumin, or polymer, have also been used clinically. Gas-filled MBs can produce strong acoustic scattering relative to the surrounding tissue. AuMBs comprise albumin-shelled microbubbles with encapsulated gold nanorods. They were investigated as a photoacoustic/ultrasound dual-modality contrast agent [48]. Wilson, *et al.*, introduced an exogenous contrastagent entitled, “triggered nanodroplets” consisting of liquid perfluorocarbon nanodroplets with encapsulated plasmonic nanoparticles that utilize vaporization for photoacoustic signal generation. Through such means, they provide significantly higher signal amplitudes than that from the traditionally used mechanism: thermal expansion. Upon pulsed laser irradiation, liquid perfluorocarbon undergoes a liquid-to-gas phase transition generating giant photoacoustic transients from these dwarf nanoparticles. Once triggered, the gaseous phase provides ultrasound contrast enhancement. Their results demonstrated, in phantom and animal studies, that photoacoustic nanodroplets can act as dual-contrast agents for both PAI and UI through optically triggered vaporization [49]. A fourth category of agents consist of biocompatible cobalt nanowontons have a Co core and an Au thin-film coating. As an aside, the name is actually derived from the Chinese eatable called the wonton. It exhibits a combination of ferromagnetic and optical responses making it amenable to dual-modality MRI and PAT studies [50]. Ma, *et al*., reported about 30 nm stable uniformly sized NIR active, superparamagnetic nanoclusters (nanoroses) formed by kinetically controlled self-assembly of gold-coated iron oxide nanoparticles. Next, small nanoclusters with optical, magnetic, and therapeutic functionality, designed by an assembly of nanoparticle building blocks, offer broad opportunities for targeted cellular imaging, therapy, and combined imaging and therapy [51]. The MPR nanoparticle is composed of a 60-nm gold core covered with the Raman molecular tag *trans*-1,2-bis(4-pyridyl)-ethylene. The thin Raman-active outer layer is protected by a 30-nm silica coating further modified with maleimide-DOTA-Gd. It was designed as a unique triple-modality MRI-PAI-Raman imaging nanoparticle (see in Figure 6) [52]. Besides these nanoparticles, Dye-loaded perfluorocarbon-based nanoparticles can also be used as PAI-FI contrast agents [21], and CdSe core/ZnS shell quantumdots can be used as PA-PT-fluorescent [40].

#### 2.1.5. Theranostic Contrast Agents

**Figure 7:**
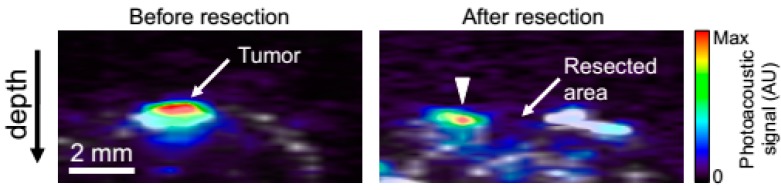
Intra-operative Photoacoustic imaging. A mouse bearing a glioblastoma tumor (primary human xenograft) was injected with MPRs (150 µL, 16 nM). After 24 h, the brain was perfused with PBS, excised, and embedded in an agarose gel. Coronal photoacoustic images were acquired before (**left** image) and after (**right** image) partial tumor resection. An absence of photoacoustic signal in the resected portion of the tumor was observed, while residual photoacoustic signal (arrow-head) was observed in the area of the non-resected tumor. Note that the increased grayscale ultrasound signal to the right of the resected cavity is likely due the surgical manipulation, an effect that is commonly observed during surgery. Photoacoustic images (color scale from 0 to max) were overlaid on conventional ultrasound images (gray), which outline the gross anatomy of the mouse brain. (Reprinted from reference [52]. Copyright with permission from © 2012, Rights Managed by Nature Publishing Group).

Exogenous contrast agent can also be used for photothermal therapy, drug therapy or image-guided surgery. In these methods, the double virtues of imaging and targeted therapy would be possessed. They can be conjuncted with antibodies, proteins, nucleic acids and peptides, or drugs cluster around lesion points. In photothermal therapy, injections are administered and the area is irradiated by laser. Following this, light energy is converted into thermal energy, which can induce a rapid rise in temperature within the tumor. The cancer cells are killed through this method while the healthy cells are left alone. Note here then that the laser then is not only the excitation light source, but also the source of treatment. Chen, *et al.*, have reported the ability of silica-coated gold nanorods to provide a stable photoacoustic signal, which implies good imaging capabilities. These nanorods thus make silica-coated gold nanorods a promising imaging and therapeutic nanoagent for photoacoustic imaging and image-guided photothermal therapy [53,54]. For the purposes of drug therapy, silver nanosystems, mentioned once above, can also be built with silica nanosphere coating whose optical extinction at near-infrared wavelengths is high and broad. Homan, *et al.*, suggest that in future designs where drugs or other therapeutic molecules are encapsulated in the silica or polymeric core, this new nano platform could provide image-guided therapy monitoring. This would open up a host of new applications where imaging and therapy are performed simultaneously [39]. Next, when Au nanocages are conjugated with anti-HER2, they are able to function effectively in drug treatments. Epidermal growth factor receptors are targeted in this technique since they overexpress on the surface of breast cancer cells (SK-BR-3). Chen, *et al.*, showed that the nanocages strongly absorb light in the NIR region, with an intensity threshold of 1.5 W/cm^2^ to induce thermal destruction to the cancer cells. In the intensity range of 1.5–4.7 W/cm^2^, the circular area of damaged cells increased linearly with the irradiation power density which suggests that this new class of bioconjugated gold nanostructures-immuno gold nanocages can potentially serve as an effective photothermal therapeutic agent for cancer treatment [62]. Kircher, *et al.*, have also designed and tested MPRs for a novel triple-modality strategy that combines MRI, photoacoustic imaging and Raman imaging. This method achieves whole-brain tumor localization for preoperative and intraoperative macroscopic delineation. It utilizes the high spatial resolution of MRI, three-dimensional imaging via PAI, and high sensitivity, specificity, and resolution of surface imaging with Raman imaging (see in Figure 7) [52]. Finally, Grootendorst, *et al.*, have observed irregularities in the distribution of superparamagnetic iron oxide (SPIO) nanoparticles within PA imaging of nodes. A decrease in contrast then correlates with metastatic involvement, as seen in MR images and histology. The results therefore show that a PA based imaging technique may be quite valuable for nodal staging in the field of surgical oncology [55].

### 2.2. Contrast Agents for TAI

**Figure 8:**
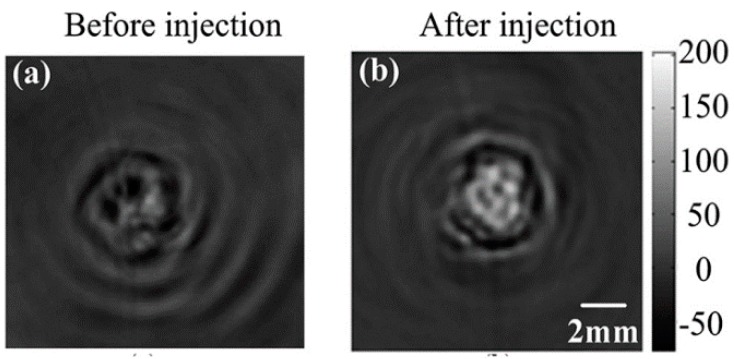
Thermoacoustic CT of tumor bearing mouse before injection of NMG_2_[Gd(DTPA)] (**a**) and after *in situ* injection of NMG_2_[Gd(DTPA)] (**b**). (Reprinted from reference [4]. Copyright with permission from © 2012 American Institute of Physics).

There are limited categories of thermoacoustic contrast agents. In this article, a detailed review of seven exogenous contrast agents for thermoacoustic molecular imaging is presented, as shown in Table 2. So far, none of them are approved for clinical applications, and their clinical applicability still needs to be properly researched. However, due to their strong magnetic field responses, efficient particle size distribution, and simple preparation, some magnetic nanoparticles have been applied in biomedicine as powerful medical diagnostic tools. The following are such examples. Carbonyl iron is one such microstructure agent. It has a size of 2 micrometers, strong microwave absorption, good hydrophilic and electromagnetic properties. It may have the potential to improve sensitivity and specificity for structural and functional TAI and broadly expand the capability of TAI [63]. Next, Dextran-coated Fe_3_O_4_ magnetic nanoparticles comprise another type of agents. They are almost spherical with diameters ranging from 30–50 nm. Qin, *et al.*, investigated the feasibility of using this nanoparticle as a contrast agent in thermoacoustic tomography (TAT) for hepatocellular carcinoma *ex vivo* detection [64]. Another set of powerful contrast agents, in biomedicine, are comprised of paramagnetic nanoparticles due to their special paramagnetism, bulk effect, and high relaxations. NMG_2_[Gd(DTPA)] is one such paramagnetic ionic compound with seven unpaired electrons in the 4f orbital of the Gd^3+^ ion. The charged ions and unpaired electrons can interact with a microwave field and transform the absorbed microwave energy into heat. Qin, *et al.*, verified the enhanced effect of NMG_2_[Gd(DTPA)] for thermoacoustic CT *in vitro* and *in vivo* experiments. They demonstrated that this contrast agent can increase the ionic conductivity and microwave absorption coefficient of tumors, thus making the boundary between tumor and normal tissue more obvious in TAI (see in Figure 8) [4].

**Table 2:** Contrast agents for thermoacoustic imaging.

Thermoacoustic Contrast Agent	Type	Excitation Source Frequency (GHz)	Size(nm)	Modification Application	Application	Ref.
Carbonyl Iron	Magnetic nanoparticles	1.2	2000		TAI, in tissue phantoms	[63]
Dextran-coated Fe_3_O_4_ Nanoparticles	Magnetic nanoparticles	6	30–50	Dextran	TAI, in tissue phantoms	[64]
NMG_2_[Gd(DTPA)]	Paramagnetic ionic compound	6			TAI, *in vitro* and *in vivo* tumor-bearing mouse	[4]
Fe_3_O_4_/Polyaniline (PANI)	Superparamagnetic nanoparticles	6	30–50	Folic Acid (FA)	*Ex vivo* TAI in human blood and *in vivo* TAT in mouse tail, *in vivo* TAI of tumors	[65]
Fe_3_O_4_ /Au Nanoparticles	Fe_3_O_4_ core/Au shell Nanoparticles	6	30–50	FITC-labeled integrinαvβ3mAb	Triple-modality MRI-TAI-PAI	[66]
Single-walled Carbon Nanotubes(SWNT)	Multimodality Contrast Agent	3	Diameter: 1.2–2.2; length: 500–1000		*In vitro*, dual-modality PAI-TAI	[67]
Microbubbles	Multimodality Contrast Agent	3	18,000		UI, and *in vitro* TAI	[68]

**Figure 9:**
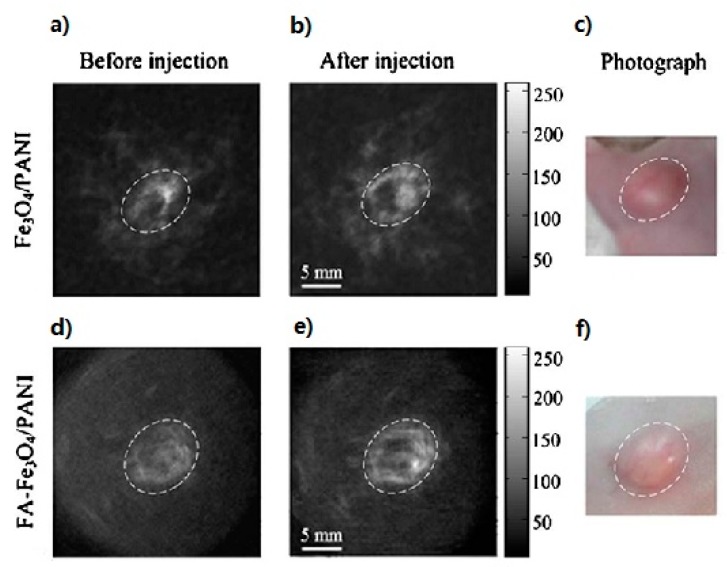
*In vivo* thermoacoustic imaging of tumors with contrast agent bearing in mouse. (**a**,**d**) TAT images of control sample injected with PBS; (**b**,**e**) TAT images of tumor injected with Fe_3_O_4_/PANI and Fe_3_O_4_/PANI-FA, respectively; (**c**,**f**) Photographs of tumor’s area on the mouse back. (Reprinted from reference [65]. Copyright with permission from © 2010 Am. Assoc. Phys. Med.).

Yet, another compound is Fe_3_O_4_/polyaniline (PANI), which is made of superparamagnetic nanoparticles conjugated to folic acid (FA). These nanoparticles can then bind specifically to the surface of the folate receptor, a tumor marker. Nie, *et al.*, have used a 6 GHz TAT system to successfully investigate that intravenous administration of the targeted nanoparticles to mice bearing tumors showed a five-fold greater thermoacoustic signal and a much longer elimination time than that of nontargeted nanoparticles.This portrays Fe_3_O_4_/polyaniline (PANI)’s potential for its use in targeted and guided cancer thermal therapy (see in Figure 9) [65].

Similarly, an Fe_3_O_4_ core can be bound to an Au shell to create Fe_3_O_4_ core/Au shell nanoparticles in order to combine the advantageous and highly complementary features of the Fe_3_O_4_ and gold nanoparticles. Zhou, *et al.*, developed the bio-modified Fe_3_O_4_/Au NPs, which generate enhanced MR contrast and high photoacoustic and microwave-induced thermoacoustic signals. Such NPs can also be used for specific targeting and fluorescent imaging when conjugated to cancer cell targeted integrin α_v_β_3_-positive cancer cells with FITC-labeled integrin α_v_β_3_ mAb [66]. Due to its multimodality properties, single-walled carbon nanotubes (SWNTs) may also be utilized for TAI. Along with their intrinsic physical properties, they have many advantages, including their ability to encapsulate medically relevant metal ions within their carbon sheath, and externally functionalize the carbon sheath with a variety of imaging agents. Pramanik, *et al.*, developed a novel carbon nanotube-based contrast agent for both thermoacoustic and photoacoustic tomography. In their study, SWNTs exhibited much more signal enhancement for thermoacoustic and photoacoustic signal than deionized water and blood. The large contrast enhancement of SWNTs was further corroborated by tissue phantom imaging studies [67]. Besides SWNTs, microbubbles are another multimodality agent that has been used in UI and PAI due to their echo enhancing properties and optical absorption properties. Microbubbles are likely to be used in TAI modalities because of their low microwave absorption. Mashal, *et al.* characterized the dielectric and acoustic properties as well as the thermoacoustic response of mixtures with a varying concentration of air-filled glass microbubbles in TAT. Microbubbles significantly lowered the microwave absorption level and increased the acoustic velocity of the target, which reduced both the magnitude and temporal width of the thermoacoustic response. The same reduction in amplitude, but a broadening of the temporal width was obtained when using organic-shell microbubbles [68].

Finally, nano-magnetic fluid is a kind of colloid consist of nano-structured ferromagnetic particles disperse in the carrier liquid via a surfactant. Ferromagnetic particles conjuncted with antibodies, sulfur functional groups, or drugs cluster around leision points after injection, followed by microwave irradiation. Electromagnetic energy is then converted into thermal energy, which can induce a rise in temperature. This kills the cancer cells while leaving healthy cells alone. Magnetic fluid hyperthermia then could potentially be a meaningful application of TAI.

## 3. Conclusions and Future Directions

Effective photoacoustic or thermoacoustic contrast agents should significantly increase contrasts, effectively improve imaging depths or accurately provide molecular specific information. It is therefore prudent to follow these principles when choosing contrast agents: (1) photoacoustic or thermoacoustic contrast agents should be easily prepared and produced for a low cost due to future clinical applications; (2) the size of contrast agents has a significant impact on their distribution and drug delivery *in vivo*. Large contrast agents remain *in vivo* longer than small ones; however, small ones have better permeability than large ones. Greater permeability makes them readily enter the bloodstream and penetrate into the desired location after injection. Dimensional control of contrast agents exerts an important control on distribution and overall curative effect of drug; (3) agents should be of excellent water solubility, or they will be treated as foreign objects and rejected by the surrounding tissue; (4) they should be of proper biological compatibility and low toxicity in order to not cause adverse reactions to the tissues—the most major being damage to the endothelium, vascular system, blood-brain barrier (BBB), kidneys, and so on; (5) contrast agent should have a tendency to bind molecular probes (such as antibodies, proteins, nucleic acids and peptides), drugs or another contrast agent. They should have good surface modification and be able to effectively target specific receptors in target cells or tissues; (6) have a high stability, long half-life, and full dissolution *in vivo* in order to make them meet clinical requirements; (7) they should possess very good dispersibility in order to distribute evenly in the imaging region; (8) photoacoustic contrast agents should have a high light-to-ultrasound conversion efficiency, while thermoacoustic contrast agents need a high microwave-to-ultrasound conversion efficiency. Contrast agents need to be prepared and used discreetly in accordance with the provisions of the Food and Drug Administration (FDA).

Despite many years of research, photoacoustic and thermoacoustic molecular imaging still has a lot of issues that need to be addressed promptly. The following future directions should be discussed: (1) There are limited categories of thermoacoustic contrast agents. Special superparamagnetism and bulk effect, the compounding of superparamagnetic nanoparticles, and their application in biomedicine will hopefully draw the attention of research fellows. Such methods show potential for the advance in multimodality technology of PAI, TAI and MRI; (2) The quantitative capability of photoacoustic and thermoacoustic molecular imaging is significant. It may help us differentiate benign from malignant lesions as well as obtain absolute tissue functional information. They are perhaps determinants of molecular typing. Besides, PAI and TAI can measure the amount of a drug at its site which will provide the theoretic foundation for ensuring the accuracy of injection doses for animals and patients; (3) The future research of contrast agents as a drug delivery system should emphasize its effectiveness and safety. The technical specifications of the system’s effectiveness and safety are not established yet. How to reduce toxicity and side effects as well as improve the drug targeting, solubility, stability, slow releasing action, and how to change tissue-targeted distribution and metabolism are key issues for this research; (4) Mono-modality imaging cannot obtain all the information required. Compared to low resolution ultrasound imaging, TAI can distinguish adsorbers which have the same acoustic impedance but different dielectric properties. PAI is also able to capture information about optical absorption with high resolution. Compared to PET and SPECT, these two imaging technologies have no risk of causing injury by radiation. Compared to MRI, PAI and TAI have quicker imaging speed. In comparison to OCT, they have longer imaging depths. Overall, PAI and TAI only demands imple equipment, have low cost, and are easy to operate, thus they have broad and applicable prospects. The combination of TAI and PAI is capable of revealing information such as water and ion concentration, blood volume, and oxygenation of hemoglobin. The preponderance complementarity of PAI, TAI, and other imaging modes will significantly improve medical high-tech, which will lead to more comprehensive sources of information about structural and functional physiology and pathology; (5) Unfortunately, the application of photoacoustic and thermoacoustic contrast agents in real time imaging and their ability to confirm lesions before treatment cannot currently meet the needs of modern medicine. Instead, targeted drug delivery systems, advanced physical therapy, image guided surgery, heat therapy (e.g., high intensity focused ultrasound [HIFU] or radiofrequency hyperthermia), photothermal therapy, and magnet fluid hyperthermia are therefore the developing directionfor the future. Photoacoustic and thermoacoustic molecular imaging aim to integrate detection, monitoring, and reasonable treatment into one entity; (6) Contrast agent testings are too complicated, due to their specific animal testing experiments, longer experiment durations followed by clinical trials and high research and development costs. It has been concluded that the related research is unsystematic, has no unified standard, and is rarely used in the clinic due to various differences in species, individuals, and drug delivery systems. Therefore, the effectiveness and safety of photoacoustic and thermoacoustic molecular imaging play a key role in establishing better and more direct relations between basic research and clinical applications.

Photoacoustic and thermoacoustic molecular imaging, working in tandem with powerful contrast agents, could provide groundbreaking opportunities for early diagnoses, precise lesion localization, molecular typing, drug delivery monitoring, image guided surgery, drug targeting therapy, and photothermic therapy of various diseases (such as cancer and cardio-cerebrovascular disorders). Photoacoustic and thermoacoustic molecular imaging, complemented by the development of such agents, will potentially bridge the gap between bench and bedside and further develop translational medicine as we know it.
